# Trial emulation and survival analysis for disease incidence registers: A case study on the causal effect of pre‐emptive kidney transplantation

**DOI:** 10.1002/sim.9503

**Published:** 2022-07-09

**Authors:** Camila Olarte Parra, Ingeborg Waernbaum, Staffan Schön, Els Goetghebeur

**Affiliations:** ^1^ Department of Applied Mathematics, Computer Science and Statistics Ghent University Ghent Belgium; ^2^ Department of Clinical Epidemiology, Biostatistics and Bioinformatics, Academisch Medisch Centrum University of Amsterdam Amsterdam The Netherlands; ^3^ Department of Statistics Uppsala University Uppsala Sweden; ^4^ Swedish Renal Registry Jönköping County Hospital Jönköping Sweden

**Keywords:** causal inference, disease registries, kidney transplantation, observational study, survival analysis, target trial emulation

## Abstract

When drawing causal inference from observed data, failure time outcomes present additional challenges of censoring often combined with other missing data patterns. In this article, we follow incident cases of end‐stage renal disease to examine the effect on all‐cause mortality of starting treatment with transplant, so‐called pre‐emptive kidney transplantation, vs starting with dialysis possibly followed by delayed transplantation. The question is relatively simple: which start‐off treatment is expected to bring the best survival for a target population? To address it, we emulate a target trial drawing on the long term Swedish Renal Registry, where a growing common set of baseline covariates was measured nationwide. Several lessons are learned which pertain to long term disease registers more generally. With characteristics of cases and versions of treatment evolving over time, informative censoring is already introduced in unadjusted Kaplan‐Meier curves. This leads to misrepresented survival chances in observed treatment groups. The resulting biased treatment association may be aggravated upon implementing IPW for treatment. Aware of additional challenges, we further recall how similar studies to date have selected patients into treatment groups based on events occurring post treatment initiation. Our study reveals the dramatic impact of resulting immortal time bias combined with other typical features of long‐term incident disease registers, including missing covariates during the early phases of the register. We discuss feasible ways of accommodating these features when targeting relevant estimands, and demonstrate how more than one causal question can be answered relying on the no unmeasured *baseline* confounders assumption.

**Abbreviations**
ATE: average treatment effect
ATT: average treatment effect among the treated
ATNT: average treatment effect among the non‐treated
ESRD: end‐stage renal disease
ITT: intention‐to‐treat
IPW: inverse probability weighting
PKT: pre‐emptive kidney transplantation
PS: propensity score
RCT: randomized controlled trial
SRR: Swedish Renal Registry

## 1 INTRODUCTION

For decades now, the randomized clinical trial (RCT) enjoyed the status of bringing gold standard evidence to inform clinical decisions.^1^ While advantages of this design are undeniable, the call for additional real world evidence sounds ever louder. This stems in part from the restricted and somewhat artificial setting of the randomized experiments, which challenges transportability of results to real world target populations.^2^, ^3^ It is further the fruit of growing data resources of various types harboring information from much broader natural target groups, which can now be mined using new developments in causal inference and beyond.

Today's evidence supporting clinical decisions is thus also drawn from observed exposures, both within randomized trials, as requested in the ICH E9 appendix on estimands,^4^ and in the absence of trials. This becomes the primary evidence source when treatment cannot be randomized due to ethical or practical reasons as is typical for organ transplantation, for instance. A well designed (comprehensive) cohort of diseased patients may then bring the best chance of obtaining real world evidence. The latter should ideally be cast in clinically interpretable measures. Hence risk differences will be preferred over hazard ratios even though the latter may be an essential vehicle to arrive at the former.

For a range of chronic diseases, population based incidence registers following patients from disease onset have been built and maintained over years. These are now important data sources for investigations of long term outcomes. Accrual over many calendar years also comes with additional challenges. Earlier entries are automatically subject to longer administrative censoring times. When patient profiles and/or general level of care changes (improves) over calendar time several consequences must be addressed. First, assuming the study population is our target population, the population average survival curve, as estimated by Kaplan‐Meier (KM) will be biased. Indeed, the longer administrative censoring times may then come with better survival chances. This informative censoring can be handled by simply adjusting for registry entry time. That well‐known fact,^5^, ^6^ gets easily forgotten given the robust reputation of the KM curve. It is not remedied by inverse probability of treatment weighting (IPW) adjustment which may address covariate imbalance across treatment groups. Second, having learned how entry time impacts survival on one or either treatment, the causal question of interest may shift from the full study population to what recent or even future patients can expect to benefit from their choice of treatment. Still, careful analysis of the available cohort will lay the foundation of such insight.

When analyzing the effect of a point exposure on survival, one will obviously need to adjust for confounders associated with treatment at the time of treatment decision. It is then a great advantage that registries at the national level with broad coverage typically have a well worked out protocol carefully defining the set of patient characteristics to be included by all centers at the time of patient entry into the registry. Naturally this set may get updated after a number of years on a given date to include additional covariates, responding to progressing insight in prognostic factors or more easy access to the (good) measurements.

What happens during later follow‐up tends to be much less controlled or harmonized as it emerges over long periods of time in a range of settings with more or less support for data measurement. Tight control would be extremely demanding at that level. It is hence important to understand what can be estimated when relying on a common set of baseline covariates without access to regular time‐varying covariate measurements.

As with clinical trials, the target estimand can either follow the intention‐to‐treat (ITT), per‐protocol or as‐treated principle addressing a corresponding causal question. In the randomized trial, the ITT analysis commonly estimates the causal effect of being *assigned to a particular treatment* regardless of the adherence to it. In the observational setting it will also pertain to a point exposure which could be controlled at a specified time of “treatment” onset common to the available treatment options, such as treatment assigned, prescribed or initiated. Here too ITT marginalizes over subsequent treatment (intensity). An appreciation of exposure levels that follow in the study population will deepen our understanding of exposure and influence transportability of the estimand.^3^, ^7^, ^8^ Of course, before comparing outcomes of treatment groups conditional on covariates in the emulated trial, explicit adjustment for baseline confounders is required. This will ensure exchangeability before averaging over a chosen distribution of baseline covariates. This could be the covariate distribution observed in the full study population (average treatment effect, ATE) in the treated (ATT), the non‐treated (ATNT),^9^ or any other relevant distribution.

A per‐protocol analysis targets the effect of adhering to a treatment regimen as established by the researcher. Strategies to deal with deviations from this regimen must then be specified. One approach restricts analysis to patients fully adhering to the treatment protocol or censors patients as they deviate from it. The latter may introduce informative censoring as time‐varying factors likely influence both the treatment path and the outcome. Therefore, besides adjusting for baseline confounders, the per‐protocol effect also requires adjusting for time‐varying confounding.

Finally, the as‐treated effect in RCTs pertains to treatment actually received (possibly for a given duration), rather than randomized to. No longer under the protection of randomization, this approach typically involves accounting for both baseline and time‐varying confounding, even in the context of an RCT.^10^

Through trial emulation, observational data can be used to mimic as closely as possible the data set‐up that would have been aimed for in a target trial designed to answer the clinical question. It helps avoid bias frequently encountered in observational studies, for example, when allowing patient eligibility to rely on information obtained after treatment onset.^11^, ^12^

In this article, we present a case study where trial emulation draws on the Swedish Renal Registry (SRR), a nationwide research register. The research question investigates the total effect on all‐cause mortality of immediate kidney transplantation vs starting with dialysis possibly followed by delayed transplantation. The complications encountered and approaches taken apply quite generally to long term disease registers beyond nephrology. In Sweden and the Nordic countries, research on long term effects of a variety of chronic diseases is conducted through linkage of incidence registers and administrative registers with individual level data, for example, the in‐hospital register held by the National Board of Health and Welfare. The resulting datasets constitute a high‐quality observational data resource for researchers used to both support and generate new hypotheses for a wide range of diseases.

In what follows we introduce our case study in more detail in Section 2, we elaborate on the targeted estimands in Section 3 to discuss the estimation approach for ITT and an as treated analysis allowing for nonrandom treatment switch while relying on a sufficient set of baseline covariates for noninformative censoring. Both approaches with their respective results are described in Sections 4 and 5, respectively. The results derived from our case study, are followed by a note on the existing software packages to aid these analyses in Section 6 and we end with a discussion on strengths and weaknesses of the approach taken and results obtained, relative to what is currently in the literature in Section 7.

## 2 CASE STUDY

As kidneys are vital organs, patients reaching end‐stage renal disease (ESRD) need treatment to survive. The two main alternatives are dialysis or kidney transplantation, collectively known as renal replacement therapy (RRT). Several studies consider how the modality of RRT impacts survival. Specifically, one aims to determine whether and by how much *patients* with immediate transplant, so‐called pre‐emptive kidney transplantation (PKT), have better *survival* than they would have after a period of dialysis possibly followed by delayed transplantation.^13^ This setting is unique to kidney transplantation compared to other organ transplants where there is not an alternative treatment and access to a transplant is the only option to survive. Thus, different considerations and approaches are needed compared to those made in previous studies focusing on lung transplant.^14^, ^15^ It is worth noting that here we are considering dialysis as possibly a bridge therapy to a delayed transplantation, where an eventual delayed transplantation is considered part of the treatment. Other studies have compared PKT with patients who started on dialysis and censor them when they received a transplant^16^ or with a transplant not being available and remaining on dialysis.^17^

**FIGURE 1:**
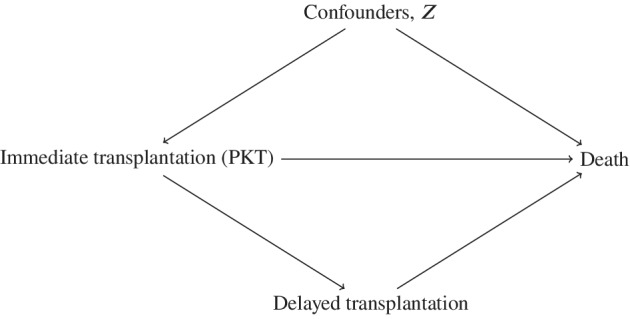
Causal diagram for the effect of immediate vs delayed transplantation on death

A systematic review of this research question^18^ identified published studies, most of them suffering from avoidable biases. Those who worked with transplant registers are limited to RRT patients receiving a transplant and obtained retrospective information on when they reached ESRD and started dialysis. The restriction to patients living long enough to obtain the transplant results in immortal time bias.^19^, ^20^ To account for this, some condition on the amount of time spent previously on dialysis lacking correction for truncation. This analysis ignores the mechanism of selecting subjects who (1) started RRT with dialysis in response to covariates then available and (2) have survived long enough to undergo transplantation.^21^, ^22^, ^23^, ^24^ Conditioning on covariates (denoted by Z hereafter) measured at the time of transplant or beyond (graft function or graft rejection) amounts to adjusting for events on the causal path from treatment initiation at RRT to survival,^25^, ^26^ another approach well known to introduce bias (Figure 1).^27^

Analyses typically start from hazards and hazard ratios whose estimation entails limited additional modeling assumptions when censoring is noninformative or explainable. Semiparametric (extended) proportional hazards models are fast and stable to implement with residual plots to help assess model assumptions. Estimation of these building blocks avoids modeling the nuisance study‐specific censoring mechanism per se. Derived summary measures, such as Z‐specific or Z‐standardized survival curves, carry direct clinical interpretation. When the models are oversimplified however (sometimes when assuming time constant hazard ratios over the long haul) or the hazards are naively interpreted as causal contrasts for populations of survivors at time t since RRT, problems arise.^28^

In what follows we overcome the problems through an alternative design, transparent analysis under clearly stated assumptions and outcome parameters chosen for clinical interpretability. An incident RRT registry allows us to mimic the ideal study which *randomizes* patients at RRT onset over either PKT or dialysis first. These exchangeable groups in terms of measured and unmeasured baseline prognostic variables are followed until death or administrative censoring. The nationwide SRR is such a cohort with carefully collected data since 1991 from all RRT units in Sweden, including 100% of transplanted patients and at least 95% of dialysis patients.^29^, ^30^ Today, it records the following covariates when patients enter the register: date of RRT onset, demographic variables at RRT onset, cause of kidney disease, RRT modality, comorbidities, kidney function and survival status.^31^ Some covariates are introduced into the registry only years after it started: comorbidities (diabetes, hypertension, ischemic heart disease, cerebrovascular disease, and peripheral artery disease) since 1998 and kidney function since 2008.

A sufficient set of measured baseline confounders justifies the assumption of “no unmeasured baseline confounding” (NUBC).^10^ Allowing various estimation strategies to account for differences between observed treatment groups in baseline characteristics, prognosis, and potential benefit from treatments. When key covariates enter the registry late, one must either limit the analyses to the period where they are available, or consider imputation typically assuming missingness at random. With the sufficient set on board, causal effect analyses strategies include outcome regression, stratification, matching with or without propensity score, IPW or a combination in so‐called doubly robust estimators. As we describe below, simple propensity score methods may not be valid in this context.

A well‐chosen contrast between Z‐specific or Z‐standardized average survival curves for the treatments considered represents the specific population average causal effect under the NUBC assumption which we formalize in Section 4.4. An ITT analysis estimates the *total* effect of treatment assignment, comparing *marginal* survival curves between arms. This effect measure naturally averages over subsequent treatments: some patients starting on dialysis may get transplanted later, while others may die or be censored before ever getting a transplant. To interpret the effect of starting with dialysis, and especially with an intention of transporting results to new populations, one will wish to acknowledge the distribution of time to transplant among the dialysis starters. This is likely dependent on country‐specific organ availability and transplant policies. Also, patients on either arm who receive a transplant may experience a graft failure requiring a new kidney or going on dialysis. The per‐protocol and as‐treated analyses must allow for nonrandom switching off the original treatment modality. This typically involves relying on time‐varying covariates which we lack in the nationwide SRR. In Sections 3 and 5, we involve accelerated failure time (AFT) models which allow for such nonrandom switch while relying on the no‐unmeasured *baseline covariates* assumption for estimation.

Our set‐up reminds of the work of Danaei et al^32^ who compare the effect on survival of initiating statins for the primary prevention of coronary disease vs not (yet). As in our case, those who do not start the treatment of interest (statins or PKT) at time t, may start later, and the ITT analysis averages over such changes in actual exposure as they naturally occur in the studied population. The “treatment” comparison is then one of immediate vs delayed treatment initiation, where the latter comes as a compound treatment of dialysis possibly followed by transplantation. This idea of a compound treatment can be seen in other settings too, like in oncology when the interest is to compare initial chemotherapy to reduce the tumor size prior to surgery, with surgery without delay.^33^

Mindful of the above considerations, we next define our estimands of interest in more detail before engaging in a well‐motivated protocol for analysis.

## 3 CAUSAL ESTIMANDS OF INTEREST

We aim to estimate the causal effect on mortality of starting RRT treatment with PKT rather than dialysis in a population eligible to receive either. Our outcome of interest, T, is time from RRT onset to death. Using the potential outcomes framework,^34^ we consider the potential survival time from RRT onwards under two alternative possible exposures: $T_{1},$ when a kidney is received without previous dialysis and $T_{0},$ when RRT starts with dialysis possibly followed by transplantation. For this to make sense, we constrain the population to those patients for which both exposures are possible in principle, thus satisfying the positivity assumption.^35^ To determine this (sub)population both statistical and clinical arguments enter, as discussed in Section 4.1.

In RCTs, an *ITT analysis* would typically estimate the total effect on time to death by comparing the survival distribution following PKT assignment, $S_{1}(t)=P(T_{1}>t)$, with the survival distribution following dialysis assignment at RRT onset, $S_{0}(t)=P(T_{0}>t)$, ignoring whether a delayed transplant follows later. The target estimand may then be any chosen contrast, for example, the difference between the survival curves $S_{1}(t)-S_{0}(t)=P(T_{1}>t)-P(T_{0}>t)$. Often one simply focuses on the hazard ratio for treatment after adjusting for baseline covariates.

In practice, we turn to our RRT incidence registry where the ability to receive PKT depends on the patient in need of treatment as well as the availability of a suitable organ.^36^ Virtually all patients receiving PKT at RRT, would technically have the option to start treatment with dialysis. The other way around is less obvious but deemed plausible to a degree. An alternative is to restrict the analysis to patients included on the waiting list, to ensure that they are all transplant candidates. However, this approach may miss patients who have a living donor and are therefore not included on the waiting list. Unfortunately, the SRR does not have this information available. Of note, Sweden has a decentralized healthcare system and there is not a standardized organ allocation system nor common criteria to define eligible recipients. Each of the four transplant centers manage their patients and waitlist differently and independently.^37^

Given all of these considerations, the average effect of PKT among the PKT‐receivers (ATT) has a more straightforward interpretation than the corresponding ATNT. Without knowing how to find a kidney for transplant at present, we may still aim to evaluate what would happen if the PKT treatment became available. This follows the philosophy on causal effects from Vandenbroucke^38^ and Hill.^39^ The ATE within the full cohort considered capable of receiving either treatment will be a weighted average of the ATT and ATNT.

A different estimand of interest may indicate the survival time lost while being treated with dialysis when waiting for a later transplant, relative to $T_{1}.$ Instead of ignoring any delayed transplant as in the ITT, it considers observed time T ($=T_{0}$) in the delayed transplantation group as a sum of two observable variables: $T=T_{w}+T_{r}$, where $T_{w}$ is the survival time spent without initial transplant and $T_{r}$, the residual survival time following the delayed transplant (if any).

The estimand may then focus specifically on the amount of time spent without transplant and estimates its effect. This would resemble the *as‐treated analysis* described by Danaei et al^32^ by focusing on the “total duration of treatment.” In our case, we model the potential survival under PKT, $T_{1}$ as a function of $T_{w}$ and $T_{r}$ as: $T_{1}\overset{d}{=}T_{w}\exp(-\psi )+T_{r}$. Every day on initial dialysis then counts as the potential exp(−ψ) days on PKT (eg, for exp(−ψ)=2, the median survival time while on initial dialysis would have been doubled under PKT treatment). This model leaves the residual survival time unaffected by an initial trajectory with or without transplant.

An alternative as‐treated model, transforms both parts of the sum to reflect an additional impact of the delayed transplant, for example, $T_{1}\overset{d}{=}T_{w}\exp(-\psi_{w})+T_{r}\exp(-\psi_{r}),$ where generally, the ψ‐parameters could depend on other baseline factors, and the timing of the transplant.

For completeness, we point to the effect of choosing to transplant at a given delay time $t_{0}$ post RRT, vs further delaying the transplant or even staying on dialysis throughout. To answer questions on the effect of transplant timing one needs, however, measures on time‐varying confounders of the timing of transplant in the dataset. Without such data, we set out to estimate the first two estimands described here, relying on the assumption of no unmeasured *baseline* confounders as explained in the following two sections.

## 4 TARGETING THE INTENTION‐TO‐TREAT ESTIMAND

In the real world, the comparison between $T_{1}$ and $T_{0}$ relies on estimating the survival of two groups of different patients with similar baseline covariates but who experienced different treatments. To allow a causal effect estimate, we will rely on the following assumptions, where Z refers to baseline covariates:

A1.
Positivity: Patients in the study population have a non‐zero, non‐one probability of receiving either treatment, given their covariate values Z: 0<P(PKT=1|Z)<1 and 0<P(PKT=0|Z)<1.
A2.
Noninformative censoring for estimation of the conditional survival function P(T>t|PKT=p,Z): censoring time, C, is independent of survival time T, given covariates {PKT,Z}:C⊥T|{PKT,Z}.
A3.
Missing at random: The missing values of the covariates are missing at random conditional on the observed covariates Z. If R denotes the missingness indicator, then P(T>t|PKT=p,Z,R=1)=P(T>t|PKT=p,Z,R=0).
A4.
No unmeasured baseline confounders (NUBC): The vector of potential survival times $\left\{T_{0},T_{1}\right\}$ is independent of observed PKT given baseline covariates Z, $\{T_{0},T_{1}\}⊥\text{PKT}|Z$. A causal effect can then be represented by contrasting $S_{1}(t;Z)=P(T_{1}>t|Z)=P(T>t|\text{PKT}=1,Z)$ with $S_{0}(t;Z)=P(T_{0}>t|Z)=P(T>t|\text{PKT}=0,Z)$.

### 4.1 Assessing positivity

To satisfy the positivity assumption (A1), the propensity score distributions of the observed treatment groups must overlap.^35^ For (sub)populations that are open to starting either treatment a meaningful population treatment effect can be estimated. Subgroups with treatment propensity (close to) zero or one obviously violate the positivity assumption as they represent groups with little chance of receiving one of the treatments. To check for this, a propensity score (PS) model was built using logistic regression for the probability of receiving PKT from baseline covariates age, sex, region, primary kidney disease and calendar year of RRT onset. Interactions between age and sex with primary kidney disease and calendar year of RRT onset were included. Notwithstanding generally good overlap of PS in both groups (Figure 3), we found that patients with a history of cancer or older than 75 years appeared to rarely receive PKT yielding a PS close to 0. We excluded them from the target population thus adjusting the scope of this analysis.

### 4.2 Informative censoring

We estimated survival from the date of RRT onset onwards censoring patients still alive by December 31, 2017, as confirmed by the cause of death registry. KM curves per treatment group present robust survival chances in selected observed treatment groups, provided noninformative censoring holds (A2). This assumption fails when conditioning on covariates Z is required to render censoring time C independent of the survival time: C⊥T|Z. This can easily happen in long term disease registers, if cohorts entering later differ in baseline prognostic factors and/or enjoy a better survival time (conditional on these baseline factors). In the early years of the registry, transplants were more risky and immunosuppression less well developed. PKT was therefore offered only to highly selected groups eg younger and healthier patients. Over time, this treatment option was extended to a broader group of patients. This structure introduces informative censoring for the unadjusted KM curves as later cohorts are censored sooner but can anticipate longer survival than similar patients who entered earlier and have longer censoring times. Aware of this problem, we start by presenting “the usual” KM curves for two observed groups: PKT and dialysis first and see already a much higher curve for PKT=1 patients, impacted however by baseline confounding and informative censoring as we explain later.

### 4.3 Selection and immortal time bias

Another source of bias enters when we consider the subset of PKT=0 patients who received delayed transplantation before study end. Figure 4 shows the overestimation of survival in the dialysis arm when studying this selective subgroup and it also suggests the extent of the immortal time bias explained in Section 2.

Further insight into the dialysis first group is supported by showing the cumulative incidence of transplant and of death without transplant. Since the vast majority of patients experienced either competing event in this arm, any remaining informative censoring becomes negligible here.

### 4.4 Handling missing covariates

Not all the envisaged confounders (age, sex, region, primary kidney disease, calendar year of RRT onset, diabetes, hypertension, ischemic heart disease, cerebrovascular disease, and peripheral artery disease) were always measured. Comorbidities were available only from 1998 onwards. Removing patients who entered before 1998 would result in losing 36% of events, a substantial information loss. Instead, we imputed the missing values for the earlier cohort assuming missingness at random (A3), effectively extrapolating their conditional distribution from 1998 (allowing for a trend in calendar time) toward the early years. Similar patterns are found in long term chronic disease registers that introduce additional covariates when the registers are already established. We decided not to impute kidney function because it was introduced later (only in 2008) and the reported measure was not standardized, that is, it is not a mandatory variable, centers can provide different measurements to report and different follow‐up time points. Instead, we assessed the impact of kidney function as part of the sensitivity analyses described in Section 4.6.

Following Clark and Altman,^40^ we included the mortality indicator and log(survival time) in the imputation model as covariates, besides age, sex, region, primary kidney disease, calendar year of RRT onset and PKT. For computational efficiency, we first created 10 imputation datasets using the R package mice^41^ and then we bootstrapped each imputed dataset to construct 95% CI using the R package boot^42^ following previous recommendations.^43^

### 4.5 Adjusting for confounders

To adjust for baseline confounders in survival analysis, one has in principle four options: regression adjustment, matching, inverse probability weighting, and/or a combination in a double robust method. We have opted for the regression adjustment because it allows us to automatically adjust for covariates known or suspected to affect censoring. For instance, by adjusting for calendar time of entry into the register we remove some informative censoring from the analysis. In contrast, inverse probability weighting would balance covariates between the treatment groups, but observed unadjusted hazards in each group would still be subject to censoring that is influenced by baseline covariates (including calendar time of entry). To correct for this we would need additional time‐varying inverse weighting for censoring. Thus, our choice here aims at simplicity and robustness for the setting. Below we describe our modeling approach which is then compared with the IPW alternative to illustrate these considerations. Even though recent work describes matching for adjustment of KM curves^44^ we have not moved forward with this option. Since the PKT group is considerably smaller than the dialysis first group, there might be difficulties in making inference for the dialysis first population using this estimator. Here, finding good matches to the dialysis first group among the PKT individuals may mean that some PKT individuals are used as matches many times, thereby inflating the variance.^45^, ^46^

We built Cox models for mortality separately in the PKT group and dialysis first group in each imputed dataset. The separate models give more flexibility, allowing for different baseline hazards and covariate effects for each treatment in a setting where there is potential for a different evolution over time. To avoid smoothing bias, we use a common set of confounders that are adjusted for in both models. We included the listed covariates as main effects and also interactions between age and sex, age and comorbidities, and sex and comorbidities. As shown by the sensitivity analyses performed, the impact of the confounder adjustment on individual survival diminishes, once the curves are averaged over the population of interest.

We then derived covariate‐specific potential survival curves under each possible treatment ($\hat{S_{1}}[t|Z_{i}]$ and $\hat{S_{0}}[t|Z_{i}]$). The average of these two curves over the whole study population was contrasted next to estimate the average treatment effect as $\hat{S_{1}}(t)-\hat{S_{0}}(t)$, with $\hat{S_{1}}(t)=\frac{1}{n}\sum_{i}\hat{S_{1}}[t|Z_{i}]$ and $\hat{S_{0}}(t)=\frac{1}{n}\sum_{i}\hat{S_{0}}[t|Z_{i}]$.^47^

We similarly averaged over the covariate distribution observed in the PKT (and dialysis first) group to estimate the average treatment effect among the treated (and non‐treated). If the model has adjusted for a sufficient set of baseline confounders, these results can be interpreted as causal effects under the potential outcomes framework for the targeted populations. We further assess residual confounding with the sensitivity analysis described below. Without relying on the no unmeasured confounding assumption (A4), we are still contrasting well defined standardized survival curves. As secondary analysis, we repeated the analyses avoiding imputation by excluding comorbidities from the set of confounders.

To compare different adjusting approaches, we use the package ipw^48^ to build inverse probability of treatment survival curves, using the same baseline confounders as described above. For each of the 10 imputed datasets, we compute the weight for each patient and then averaged over the 10 sets to get the individual weight that was finally used in the curves.

### 4.6 Sensitivity analyses

Our approach naturally involves three types of “untestable” assumptions, namely: noninformative censoring (A2), noninformative missingness (A3), and no unmeasured confounding (A4). We consider the plausibility of each of these assumptions in turn and perform sensitivity analyses when questions arise, as described below.

#### 4.6.1 Noninformative censoring

Noninformative censoring (A2), T⊥C|{PKT,Z} is required for the hazard based survival analysis (causal or not) and defined in function of the (baseline) covariates conditioned upon. We argue that the PKT cohorts who entered the registry in more recent calendar years may have better survival because the transplant treatment conditions (eg, immunosuppression) generally improved over the years. In response the risk profiles of PKT patients entering the cohort also changed over time. Older patients were allowed to enter the PKT arm in later decades, and still survival overall improved substantially in that arm. Hence any analysis which fails to adjust for calendar time (or a sufficient proxy) may suffer from bias due to informative censoring. To illustrate the impact here, we compared the “nonparametric” KM curve, ignoring baseline covariates, with a standardized curve (averaging over covariate adjusted survival). We anticipate that the latter curve will demonstrate better survival as it is less dominated by the early cohort entries which have the longer follow‐up time and add more events to our study. We note that a KM curve on the ipw weighted data, where the weights which may involve predictors of survival time, does not remedy for this as we will explain in Section 4.7 and could make things even worse (as we found out). The IPW version may benefit from using time‐varying weights but, as already stated, time‐varying confounders are not available in the registry.

#### 4.6.2 Covariates missing at random

Our analyses rely on missingness at random (A3) for the multiple imputation approach to be valid. An alternative approach is to limit the assessment to the full cases. Thus, we considered the initial set of potential confounders on the complete cases dataset: patients who started RRT in 1998 or later whose comorbidities are registered and derive their standardized survival curves. We then examined how the standardized estimates for this subgroup change when comorbidities are dropped from the covariate list.

#### 4.6.3 No unmeasured confounding

Regarding the no unmeasured confounding assumption (A4), we are limited to what is registered and since when. Prognostic factors identified in previous studies, as comorbidities and kidney function are not available for the full cohort but only introduced in 1998 and 2008, respectively.^21^, ^23^ We have chosen to check the impact on the targeted marginalized survival curves of adding these or not—using the data in the respective calendar windows where they are available. This resulted in involving comorbidities in our analyses, after imputing them for the 1991 to 1998 period, but ignoring the GFR for our full cohort analyses.

To consider unmeasured confounders, we looked at the strength that one additional unmeasured confounder would need to have in order to qualitatively change the current conclusion. First, we repeated the estimation procedure on a subsample for which we have access to registered baseline kidney function. These new models included the covariates listed before plus kidney function as main effect and as interaction with age and sex. We then compared the survival estimated effect, with the effect derived from models that drop age as a covariate, given that age is a well‐known prognostic factor.

### 4.7 Results

**FIGURE 2:**
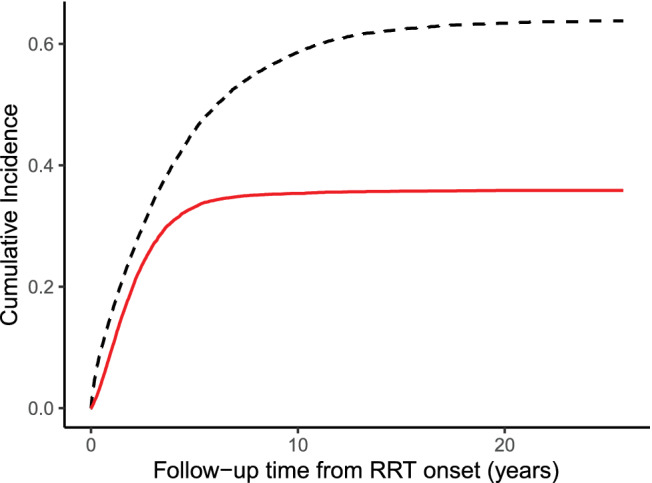
Cumulative incidence of transplantation and death without transplantation in the dialysis first group

By December 2017, the SRR included 29 526 adult patients of whom 1214 started with PKT. After excluding patients older than 75 years, non‐Swedish residents, those who received RRT abroad, and those who died on the day of RRT onset or had a history of cancer, the study population included 1097 PKT and 18 434 dialysis first patients (Table 1). The median time on initial dialysis, prior to transplant, death or censoring, was 2 years (Figure 2). There were more deaths observed in the dialysis first compared to the PKT group. Table 2 summarizes the survival outcomes.

**TABLE 1:** Study population selection and number of individuals related to exclusion criteria

	PKT	Dialysis first	Dialysis and transplant
Number of adult patients from SRR 1991 to 2017	1214 (100.0)	28 312 (100.0)	6399 (100.0)
Number of patients older than 75 years	4 (0.3)	7108 (25.1)	9 (0.1)
Number of patients from foreign or unknown region	18 (1.5)	170 (0.6)	43 (0.7)
Number of patients who receive RRT abroad	57 (4.7)	79 (0.3)	79 (1.2)
Number of patients who died or got censored on same day of RRT onset	1 (0.1)	20 (0.1)	0 (0.0)
Number of patients with a history of cancer or unknown	37 (3.0)	2501 (8.8)	234 (3.7)
Total sample	1097 (90.4)	18 434 (65.1)	6034 (94.3)

**TABLE 2:** Survival summary

(Sub)population	Patients, n (%)	Deaths, n (%)	% deaths per row	Median person‐years at risk	Hazard rate
RRT cohort	19 531 (100)	12 073 (100)	61.8	4.1	0.10
PKT group	1097 (5.6)	196 (1.6)	17.9	7.6	0.02
Dialysis first group	18 434 (94.4)	11 877 (98.4)	64.4	3.9	0.11

Table 3 describes covariate distribution in the overall RRT cohort and the PKT and dialysis first groups. PKT patients were younger and had less comorbidities than dialysis first patients. The distribution of primary kidney disease also differed between the groups. Hence, the need to adjust for confounding.

**TABLE 3:** Covariate distributions over the observed treatment groups

Covariate	RRT (n=19 531)	1. PKT (n=1097)	2. Dialysis first (n=18 434)	Difference between 1 and 2 (95% CI)
Age, median (IQR)	60 (20)	47 (22)	61 (19)	−12 (−13, −12)
Sex (female), n (%)	6867 (35.2)	411 (37.5)	6456 (35.0)	2.44 (−0.6, 5.4)
Region (Stockholm, reference), n (%)	3649 (18.7)	193 (17.6)	3456 (18.7)	−1.15 (−3.5, 1.2)
Region (Uppsala/Orebro), n (%)	4504 (23.1)	249 (22.7)	4255 (23.1)	−0.38 (−3.0, 2.2)
Region (Northern), n (%)	2017 (10.3)	95 (8.7)	1922 (10.4)	−1.77 (−3.5, 0.0)
Region (Southern), n (%)	3466 (17.7)	179 (16.3)	3287 (17.8)	−1.51 (−3.8, 0.8)
Region (Southeastern), n (%)	2376 (12.2)	127 (11.6)	2249 (12.2)	−0.62 (−2.6, 1.4)
Region (Western), n (%)	3519 (18.0)	254 (23.2)	3265 (17.7)	5.44 (2.8, 8.0)
Kidney disease (Diabetic nephropathy, reference), n (%)	5656 (29.0)	183 (16.7)	5473 (29.7)	−13.01 (−15.4, −10.7)
Kidney disease (Glomerulonephritis), n (%)	3508 (18.0)	328 (29.9)	3180 (17.3)	12.65 (9.8, 15.5)
Kidney disease (Uremia of unknown cause), n (%)	2063 (10.6)	116 (10.6)	1947 (10.6)	0.01 (−1.9, 1.9)
Kidney disease (Polycystic kidney disease), n (%)	1538 (7.9)	165 (15.0)	1373 (7.4)	7.59 (5.4, 9.8)
Kidney disease (Pyelonephritis), n (%)	640 (3.3)	41 (3.7)	599 (3.2)	0.49 (−0.7, 1.7)
Kidney disease (Other), n (%)	6126 (31.4)	264 (24.1)	5862 (31.8)	−7.73 (−10.4, −5.1)
Hypertension,^a^ n (%)	15 520 (79.5)	832 (75.8)	14 688 (79.7)	−3.8 (−4.7, −3.0)
Diabetes,^a^ n (%)	7405 (37.9)	202 (18.4)	7203 (39.1)	−20.7 (−21.5, −19.9)
Ischemic heart disease,^a^ n (%)	5196 (26.6)	49 (4.4)	5147 (27.9)	−23.5 (−23.9, −23.0)
Peripheral artery disease,^a^ n (%)	2582 (13.2)	33 (3.0)	2550 (13.8)	−10.8 (−11.2, −10.5)
Cerebrovascular disease,^a^ n (%)	2072 (10.6)	22 (2.0)	2050 (11.1)	−9.1 (−9.4, −8.8)
Outcome: Deaths, n (%)	12 073 (61.8)	196 (17.9)	11 877 (64.4)	−46.5 (−49, −44.1)

a Imputed covariates. The mean over the 10 imputed datasets is presented.

**FIGURE 3:**
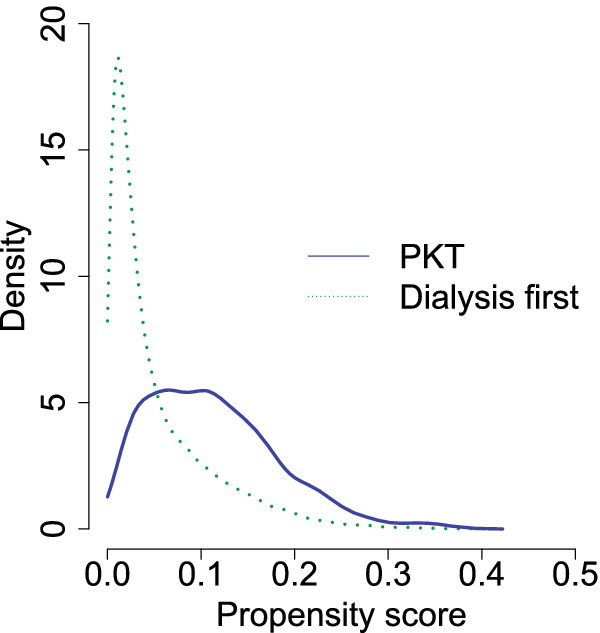
PKT propensity score density plot in the two observed treatment groups in the final study population

To assess the positivity assumption (A1), we built a PS score model for PKT. Figure 3 shows overlap in the PS for PKT between patients who effectively receive PKT and those who started on dialysis.

**FIGURE 4:**
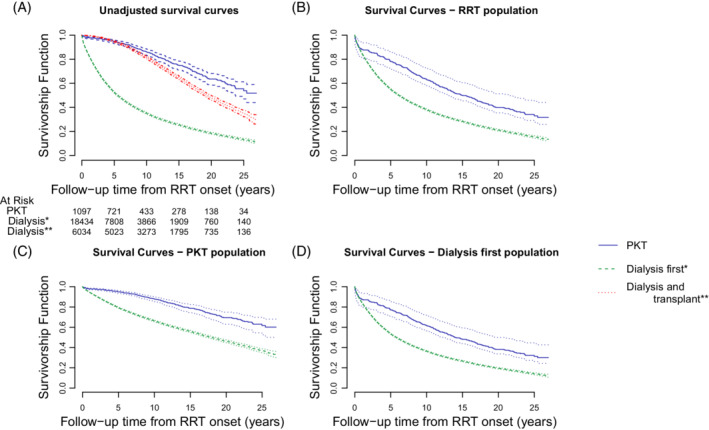
Unadjusted and adjusted survival curves from RRT onset. (A) Unadjusted Kaplan‐Meier curves of observed mortality are shown for the PKT subpopulation (blue), the dialysis first subpopulation (green) and the subset of the dialysis first who received a transplant (red). These Kaplan‐Meier curves are typically seen as robust estimates of the underlying population survival chances. Here, however, we discovered that calendar time of study entry predicts mortality and hence the unadjusted curves suffer from informative censoring. (B) Model based survival curves under each potential treatment given the full RRT population covariates. (C) Model based survival curves under each potential treatment given the PKT subpopulation covariates. (D) Model based survival curves under each potential treatment given the dialysis first subpopulation covariates

In Figure 4A, unadjusted KM curves from RRT onwards for the PKT and dialysis first group show better survival in the observed PKT group over dialysis first (log‐rank test P<.001). Since calendar time of study entry predicts mortality as described in Section 4.2, these unadjusted curves suffer from informative censoring however. In addition, the curve for the subset of dialysis first patients who were seen to receive a later transplant suffers from immortal time bias. It is dramatically shifted upwards leaving no apparent difference with the PKT‐curve for the first 5 years. Estimating survival on this selective subset clearly leads to overestimation of survival in the dialysis arm.

To account for baseline confounders, standardized survival curves were built. Figure 4 shows patient survival after RRT onset, derived from the corresponding models as described in Section 4.4: once averaged over the covariates of the whole RRT cohort and then over the PKT and the dialysis first groups separately. The PKT survival advantage over the dialysis first strategy, appears only partially explained by differences in baseline variables, for example, being younger and healthier.

Table 4 summarizes patient survival in each (sub)population at different time points. The population specific risk differences reveal dramatical treatment impact over the PKT subpopulation, the dialysis first subpopulation and the full RRT population. For the RRT and the dialysis first populations, the biggest estimated difference in survival occurs between 5 and 10 years after RRT onset. For the PKT group this difference continues to increase over follow‐up time. PKT patients form a highly selective group that enjoys better survival under either treatment.

**TABLE 4:** Survival probabilities for the different (sub)groups of interest under the two potential treatments: Pre‐emptive kidney transplantation (PKT) and dialysis first derived from the model with imputed covariates

Year(s) after RRT onset	Survival under PKT (95% CI)	Survival under dialysis first (95% CI)	Difference in survival (95% CI)
*RRT population*			
1	0.88 (0.81, 0.94)	0.85 (0.84, 0.85)	0.03 (−0.04, 0.09)
5	0.78 (0.72, 0.86)	0.55 (0.54, 0.56)	0.23 (0.17, 0.31)
10	0.63 (0.58, 0.73)	0.38 (0.38, 0.39)	0.25 (0.20, 0.35)
15	0.50 (0.45, 0.60)	0.28 (0.28, 0.29)	0.22 (0.17, 0.32)
20	0.40 (0.35, 0.51)	0.21 (0.20, 0.22)	0.19 (0.14, 0.30)
25	0.33 (0.28, 0.45)	0.15 (0.14, 0.16)	0.18 (0.12, 0.30)
*PKT subpopulation*			
1	0.98 (0.97, 0.99)	0.94 (0.94, 0.95)	0.03 (0.03, 0.04)
5	0.95 (0.94, 0.96)	0.79 (0.79, 0.80)	0.16 (0.14, 0.17)
10	0.88 (0.86, 0.90)	0.66 (0.65, 0.67)	0.22 (0.19, 0.24)
15	0.79 (0.74, 0.82)	0.56 (0.55, 0.57)	0.23 (0.18, 0.27)
20	0.70 (0.63, 0.75)	0.46 (0.45, 0.48)	0.23 (0.17, 0.29)
25	0.62 (0.53, 0.69)	0.37 (0.35, 0.39)	0.25 (0.16, 0.33)
*Dialysis first subpopulation*			
1	0.88 (0.80, 0.94)	0.84 (0.84, 0.85)	0.03 (−0.04, 0.09)
5	0.77 (0.71, 0.85)	0.54 (0.53, 0.54)	0.24 (0.17, 0.32)
10	0.62 (0.57, 0.72)	0.36 (0.36, 0.37)	0.25 (0.20, 0.35)
15	0.48 (0.44, 0.59)	0.27 (0.26, 0.27)	0.21 (0.17, 0.32)
20	0.38 (0.34, 0.50)	0.20 (0.19, 0.20)	0.19 (0.14, 0.30)
25	0.31 (0.26, 0.44)	0.14 (0.13, 0.15)	0.17 (0.12, 0.30)

Table 5 summarizes the patient survival in each (sub)population at different time points when estimated from the models built as secondary analysis, that is, they did not include comorbidities as confounders. Compared to the estimates from the full original model, the estimated survival under dialysis first for the different (sub)populations is essentially the same. However, the estimated potential survival under PKT for the dialysis first and RRT groups is slightly higher from these models compared to the estimates derived from the models including comorbidities.

**TABLE 5:** Survival probabilities for the different (sub)groups of interest under the two potential treatments: Pre‐emptive kidney transplantation (PKT) and dialysis first derived from the model without comorbidities

Year(s) after RRT onset	Survival under PKT (95% CI)	Survival under dialysis first (95% CI)	Difference in survival (95% CI)
*RRT population*			
1	0.92 (0.87, 0.95)	0.85 (0.84, 0.85)	0.07 (0.02, 0.10)
5	0.82 (0.78, 0.87)	0.55 (0.54, 0.56)	0.27 (0.23, 0.32))
10	0.67 (0.62, 0.72)	0.38 (0.38, 0.39)	0.28 (0.23, 0.34)
15	0.52 (0.46, 0.58)	0.28 (0.28, 0.29)	0.23 (0.18, 0.30)
20	0.41 (0.35, 0.48)	0.21 (0.20, 0.22)	0.20 (0.14, 0.27)
25	0.33 (0.27, 0.41)	0.16 (0.14, 0.17)	0.18 (0.11, 0.26)
*PKT subpopulation*			
1	0.98 (0.97, 0.99)	0.94 (0.94, 0.94)	0.04 (0.03, 0.05)
5	0.95 (0.94, 0.96)	0.78 (0.78, 0.79)	0.17 (0.15, 0.18)
10	0.88 (0.86, 0.90)	0.65 (0.64, 0.66)	0.23 (0.20, 0.25)
15	0.78 (0.74, 0.82)	0.55 (0.54, 0.56)	0.23 (0.19, 0.27)
20	0.69 (0.63, 0.74)	0.46 (0.44, 0.47)	0.23 (0.17, 0.29)
25	0.61 (0.53, 0.68)	0.37 (0.35, 0.39)	0.25 (0.16, 0.32)
*Dialysis first subpopulation*			
1	0.91 (0.87, 0.95)	0.84 (0.84, 0.85)	0.07 (0.02, 0.10)
5	0.82 (0.77, 0.86)	0.54 (0.53, 0.54)	0.28 (0.23, 0.33)
10	0.65 (0.60, 0.71)	0.37 (0.36, 0.37)	0.29 (0.24, 0.35)
15	0.50 (0.45, 0.57)	0.27 (0.26, 0.28)	0.23 (0.18, 0.30)
20	0.39 (0.34, 0.47)	0.20 (0.19, 0.21)	0.19 (0.14, 0.27)
25	0.32 (0.25, 0.40)	0.14 (0.13, 0.15)	0.17 (0.11, 0.26)

**FIGURE 5:**
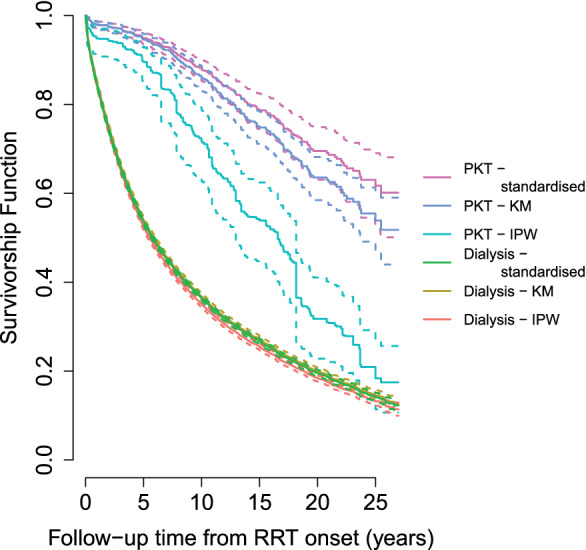
Comparison of unadjusted and adjusted survival curves through standardization and IPW

Figure 5 shows unadjusted and adjusted survival curves for the PKT and dialysis population using standardization and IPW. For the dialysis group, the curves overlap. For the PKT group, standardization yields better survival than the unadjusted KM curves, which in turns exceeds the estimated survival with IPW. This is explained as follows.

- The Cox models revealed how survival chances on the PKT group improved over calendar time of entry (one knows that better immunosuppression is one of the contributors of improved post transplant survival over the decades). As a result: (1) patients entering later with PKT enjoy better survival (given similar covariates) and (2) one allowed more frail (older and sicker) patients into the PKT treatment group in later decades. The unadjusted K‐M curves are unbiased only when censoring is uninformative without conditioning on covariates Z. In our setting, however, the unobserved future of the later entries with shorter administrative censoring in the PKT arm gets effectively (but erroneously) informed by the observed future of historical entries where subjects are followed longer and have worse survival under PKT. As a result, the K‐M curves show lower survival than they should for their study population.
- When IPW is applied to balance baseline covariates between treatment arms, it will upweight older ages in the PKT group since old age was more rare in that group. These older patients in the PKT arm appear however more in recent decades, hence with the shorter censoring times. The problem of informing their unknown future with survival chances that are too low (as explained in the previous paragraph) thus gets aggravated (as it happens for more (up‐weighted) patients).
- The survival chances in the dialysis first arms have not changed that much over the decades. In addition, the covariate distribution of entering patients is quite stable in the dialysis first arm. For both these reasons the administrative censoring in this group stays fairly noninformative and the differently constructed marginal survival curves almost coincide.

**FIGURE A3:**
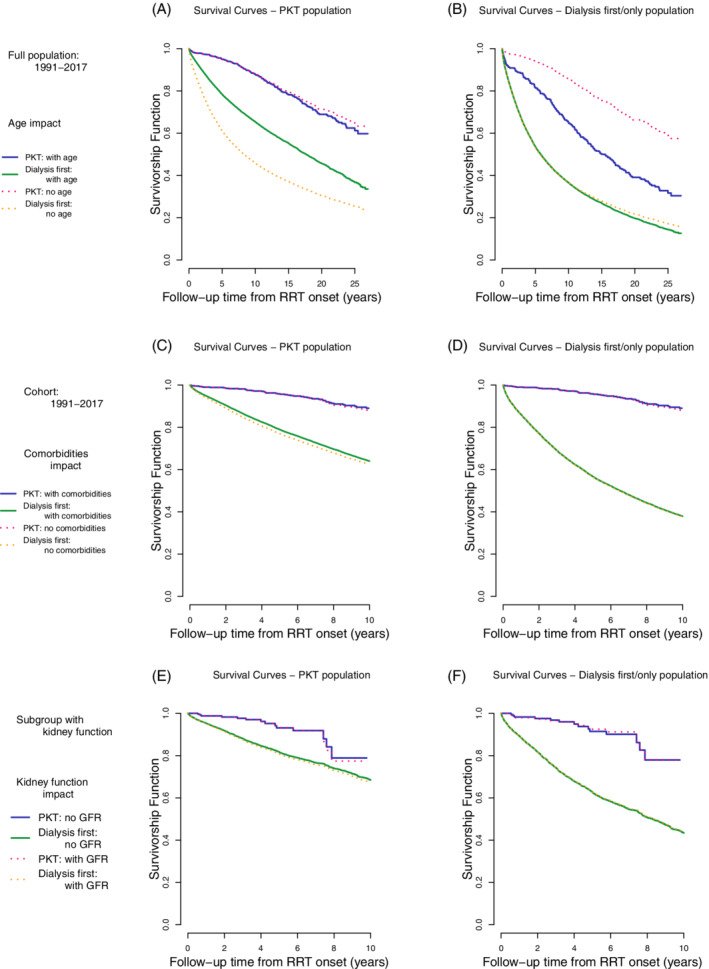
Visualizing the impact on standardized survival of including additional covariates

Figure A3A,B, in Appendix A, shows the standardized survival curves when omitting age from the original models for both the PKT and dialysis first groups as a sensitivity analyses. The curves are quite different particularly when estimating the counterfactual outcome, that is, the treatment that was not observed. However when this was repeated for the subset of patients that had additional covariates, first for those with comorbidities reported (Figure A3C,D) and then for those with baseline kidney function (Figure A3E,F) there was quite an overlap with the estimated survival curves without these additional confounders suggesting little impact of omitting such variables (Appendix A).

## 5 TARGETING THE AS‐TREATED ESTIMAND

Patients starting on dialysis continue with it for different lengths of time before possibly switching to transplant. In this section, we outline how to estimate the impact on survival of the time spent on initial dialysis. Here we make the same assumption of positivity (A1) and no unmeasured confounders at baseline (A4) as in Section 4.

### 5.1 Time lost while on dialysis

**FIGURE 6:**
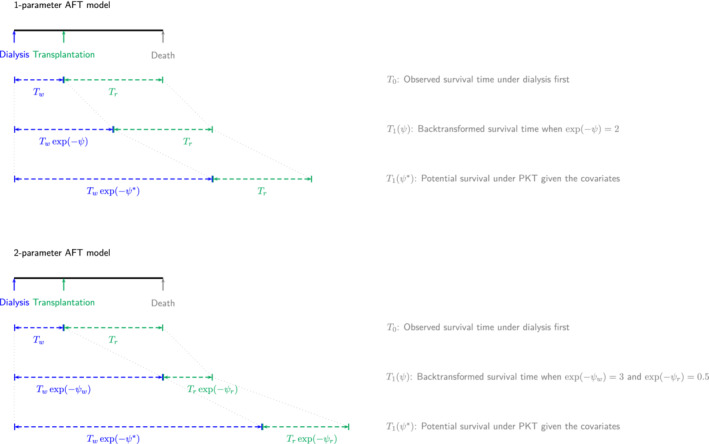
Structural accelerated failure model that relates survival time on dialysis to what it might have been following PKT. ψ represents different parameter values used to transform the survival time under dialysis to what it might have been under PKT, where $\psi^{*}$ is the true parameter

To estimate the impact on survival of the time spent on initial dialysis we invoke the structural AFT model illustrated in Figure 6.

For patients starting on dialysis, we write survival time T as the sum of $T_{w},$ the initial time spent without transplant, and a residual time $T_{r}$ post a possible delayed transplant at $T_{w}.$ Our one parameter AFT model transforms the initial time $T_{w}$ to what it might have been under PKT, and leaves the residual time that followed a delayed transplant unchanged. Specifically, $T_{w}$ is multiplied by a factor exp(−ψ) and then adds the observed time post transplant $T_{r}$ to arrive at $T_{1}(\psi ),$ the potential survival time from RRT onset to death under PKT:

$$T_{1}(\psi )\overset{d}{=}T_{w}\exp(-\psi )+T_{r}.$$

The model expresses that a day on initial dialysis would have amounted to exp(−ψ) days had the patient received PKT instead. A longer survival time under the PKT scenario corresponds to a negative value of ψ.

More generally

$$T_{1}(\psi )\overset{d}{=}\int_{0}^{T}\exp[-\psi D(u)]du,$$

where D(u) indicates whether at time u the patient is still on initial dialysis (1) or not (0).^49^ Clearly, for patients observed on PKT, $T_{1}(\psi )=T$, no transformation is necessary.

For patients starting with dialysis this model assumes that the “transplant effect” is the same whether it occurs before or after dialysis. To relax this assumption, we can introduce a second parameter to the model. This is still a simple but more flexible model that allows for an altered “transplant effect” when it is delayed after a period on dialysis rather than an immediate PKT:

$$T_{1}(\psi )\overset{d}{=}T_{w}\exp(-\psi_{w})+T_{r}\exp(-\psi_{r}),$$

where the factor $\exp(-\psi_{r})$ now backtransforms residual time after transplant, $T_{r}$, in addition to the factor for “immediate transplant effect” of PKT, $\exp(-\psi_{w})$, that multiplies the time without dialysis $T_{w}$ as in Figure 6.

Parameters in this model can be obtained through G‐estimation relying on the NUBC assumption (A4).^50^ Upon transforming the observed initial time on dialysis $T_{w}$ (and possibly also $T_{r}$) using possible values of ψ to what it would have been under PKT we obtain potential survival times $T_{1}(\psi )$. For the true parameter $\psi^{*}$ the transformed potential time $T_{1}$ $\overset{d}{=}T_{1}(\psi^{*})$ no longer depends on the observed treatment PKT, once the necessary baseline confounders have been accounted for. To estimate this true parameter $\psi^{*}$, we tested different values of ψ over a fine grid. For each possible ψ value we fitted a Cox PH model, regressing T(ψ) on Z with an additional effect of PKT on the log hazard scale: $\lambda_{T_{1}(\psi )}(t|\text{PKT},Z)=\lambda_{0}\exp\beta_{Z}(\psi )Z+\beta_{\text{PKT}}(\psi )\text{PKT}$. The value $\hat{\psi }$ for which the derived coefficient ${\hat{\beta }}_{\text{PKT}}(\hat{\psi })$ is zero is our point estimate.

A Wald test for $H_{0}:\beta_{\text{PKT}}(\psi )=0$ was used to identify the value of the parameter vector ψ with 95% confidence intervals. For the 2 parameter case, the estimation model involved an interaction between PKT and sex: $\lambda_{T_{0}}(t|\text{PKT},Z)=\lambda_{0}\exp\beta_{Z}Z+\beta_{\text{PKT},1}\text{PKT}+\beta_{\text{PKT},2}\text{PKT}\times \text{Sex}$. Here we used a Wald test statistic for the 2 estimated parameters $\hat{\beta }=\left(\begin{array}{c} {\hat{\beta }}_{\text{PKT},1}\\ {\hat{\beta }}_{\text{PKT},2} \end{array}\right)$ as ${\left(\hat{\beta }(\psi )-0\right)}^{T}[I(\hat{\beta }(\psi ))](\hat{\beta }(\psi )-0)$, where I() corresponds to the information matrix.

Note that in practice, we observe D=min(T,C) and hence calculate backtransformed observation time D(ψ)=min(T(ψ),C(ψ)), with $C(\psi )=C_{w}\exp(-\psi )+C_{r}$ defined in parallel with $T(\psi )=T_{w}\exp(-\psi )+T_{r}$. Even when C⊥T|Z, C(ψ) may depend on T(ψ) given Z. This is so, because C(ψ) depends on the time to transplant. Indeed, $T_{w}=C_{w}$, and this time of switching to transplant may be predictive of future survival, hence informative conditional on Z.

When the survival times are thus backtransformed, informative censoring is introduced because the backtransformed censoring times depend on the switch times which themselves may be prognostic for survival.

To remove this link with the switch times, we repeated this estimation process using artificial censoring on the backtransformed time scale after a common duration observable for all (the minimum transformed censoring time over all patients who started on dialysis to avoid informative censoring).^51^

**FIGURE 7:**
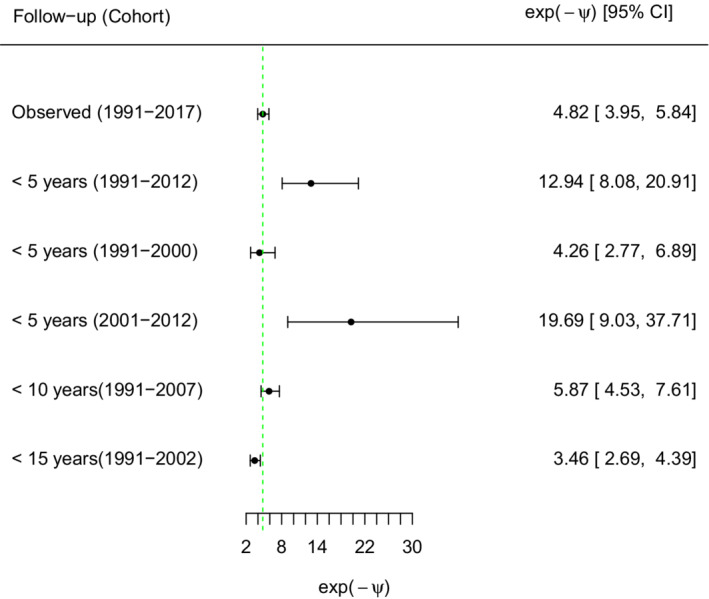
Estimated backtransformation factors for survival time under initial dialysis based on different cohorts and different windows of follow‐up time

To look into the possible impact of length of follow‐up time (considering the time‐constant hazard ratio model used within treatment groups) and the impact of calendar time of entry (or cohort), we derived Figure 7. It shows what is expected to happen in the first 5, 10, or 15 years post RRT by artificially recensoring the potential outcome data under PKT (ie, back transformed data) at those times before running the analysis. We also limit ourselves there to several calendar cohorts starting in 1991 or 2001. The figure thus illustrates the finding that the estimated treatment effect becomes larger in more recent years when the PKT treatment becomes more effective. Similarly, the wider the cohort that starts from 1991, the more the treatment effect shifts toward higher hazard ratios.

### 5.2 Results

A structural accelerated failure model with one parameter was built to account for time under dialysis. The corresponding acceleration parameter was $\exp(-\hat{\psi })$ = 4.8 (95% CI 3.9, 5.8). Therefore assuming no unmeasured confounding (A4), the survival time after PKT was almost 5‐fold the survival time while on dialysis.

Figure 7 shows the impact on the estimated AFT effect, $\exp(-\hat{\psi }),$ of different windows of follow‐up time for the potential PKT survival time (ie, backtransformed survival time) and different calendar time periods of entry (cohorts). The data were limited to those periods for this purpose. First, it is clear that the estimated $\exp(-\hat{\psi })$ for the different cohorts and chosen time windows of follow‐up consistently suggest a survival advantage of PKT. Next, the figure is consistent with the finding that the treatment effect becomes larger in more recent years (of entry): later cohorts show more effect, that happens when they start later and also when the cohort that starts in 1991 covers a broader calendar time period. There is little indication that the longer internal follow‐up time (post RRT) comes with a shifted treatment effect as long as the years of entry remain constant. In terms of precision, the shorter the follow‐up period, the wider the CI in line with fewer observed events.

Note that since our AFT effect prolonged the observed times, the backtransformed censoring times always exceeded the original ones and none of the observed events were recensored for this reason.

A second structural accelerated failure model with two parameters was built to account both for time under dialysis and beyond delayed transplantation. The corresponding acceleration parameters were $\exp(-{\hat{\psi }}_{w})$ = 5.6 (95% CI 5.1, 6.6) and $\exp(-{\hat{\psi }}_{r})$ = 0.7 (95% CI 0.5, 0.9). It is estimated that the initial time under dialysis would have been 5.6 times longer under PKT, while the additional time beyond this point would have been 30% shorter. The idea is that the transplant that happens later for people who started on dialysis may be more effective for them at that point, than the continued effect on the hazard after already a longer term post transplant.

## 6 USING AVAILABLE SOFTWARE

Existing software facilitates the application of both standardization and IPW but may not fit one's set up. The R package “stdReg” implements standardization using Cox models.^52^ The population that it standardizes the estimates to, should be a subset of the one used to fit the regression model, which is not our case. Even if we were willing to fit a single model and include treatment as a covariate and “lose” the flexibility of having different covariate effects, the package does not allow for strata which means that we would also have to assume the same baseline hazard under each treatment.

As already stated, we use the R package ipw to fit the IPW curves.^48^ However when deriving the weights, the model can have at most 20 covariates. This was exceeded with the number of covariates and interactions we adjusted for. Therefore, we evaluated the use of different subsets of the covariates included in the main model, keeping the main effects and including different interactions each time to complete the maximum of 20 covariates adjusted for. The different combinations yielded similar results as the ones already described (data not shown). We decided to present these curves as they were useful to illustrate that IPW curves may not be valid in this context due to informative censoring and lack of measured time‐varying confounders.

## 7 DISCUSSION

This case study showed potential and limitations of exploiting a nationwide incident disease register with *baseline* covariates measured across centers to produce real world evidence on PKT and its effect on mortality of ESRD patients. It demonstrated how such registers more generally enable estimation of the total effect of a well‐defined point exposure on survival time, provided a sufficient set of baseline confounders for the point exposure has been measured across the registry. Under additional semiparametric modeling assumptions, we further estimated the effect of observed time spent off‐initial‐treatment allowing the “on switch” to be affected by unmeasured time‐varying covariates. We saw that careful consideration of the target estimand has major importance as results differed vastly over the relevant options. We finally presented estimation approaches that are well adapted to the setting and discussed their assumptions in context.

Specifically, this case study has quantified to what extent PKT comes with higher standardized survival over the period 1991 to 2017 for the Swedish AT(N)T (sub)populations considered. Assuming no unmeasured confounding at baseline (A4), this represents a causal difference in survival chances. Prognostic factors differed greatly over the observed treatment groups (PKT vs dialysis first at ESRD). With all available confounders in a relatively simple Cox model, standardized marginal survival revealed a large potential survival benefit in both the observed PKT and dialysis first populations. A more complex model could have been fitted, but we saw little impact of this for our outcome.

In our analyses, we use hazard functions of the observed survival times, following either PKT or dialysis first, to model associated survival chances under either treatment conditional on measured baseline confounders (for treatment and censoring). We derive standardized survival curves from this, which represent the potential survival chance under either treatment in a well‐defined (common) population. These standardized population distributions of the potential survival times are then compared between treatments to give us a causal effect measure. A popular overall summary statistic for the contrast between these (standardized) survival curves is the ratio of their hazard functions characterizing, for which typically, a time‐averaged summary is reported. It is a causal contrast in this sense. This does NOT mean that at a given time point t past the point exposure the surviving subpopulations under PKT and under dialysis first are still exchangeable (conditional on their baseline covariates, measured at time t=0). Since different survivor selections may have taken place by then on the two treatments and switching treatments at that time would *not* correspond to switching between the established hazards for the distribution of the potential survival times.

In the PKT group (Figure 5), a striking difference emerged between the adjusted survival curve obtained through Cox regression and through the corresponding IPW weighted KM curve. This was explained (in part) by a calendar time trend in baseline risk as well as measured risk profiles, a common phenomenon in long term disease registries with complex treatments. Later cohort entry (RRT onset) translates into shorter administrative censoring time, while later cohorts show more older patients with additional comorbidities receiving PKT. IPW weighted KM curves work with covariate balance between treatment groups, but still rely on the hazard beyond censoring being well represented by that of uncensored patients. Since noninformative censoring does not hold, we must expect underestimated survival. The bias is greater even than with unweighted KM‐curves as IPW upweights patients with older age who entered later into the registry and were therefore censored sooner. The IPW approach could work when a sufficient set of time‐varying covariates were measured regularly to model the hazard of censoring and additional inverse time‐varying probability of censoring were applied. It would then of course involve a second model besides the propensity of treatment model. It is also extremely demanding and costly to measure regular time‐varying covariates across the nation, and hence unrealistic in this case.

Not only risk profiles of patients entering over time, but also treatment strategies are evolving. In our context surgical techniques were refined and newer and better immunosuppression therapy was introduced. When it comes to predicting benefits for future patients, extrapolation should incorporate these trends.

In the AFT analysis, we assessed the impact of immediate transplant vs delayed transplant on patient survival by estimating the time lost under initial dialysis. This provides further evidence that survival under PKT is better, regardless of the time under initial dialysis. Interestingly, for the AFT with two parameters, each parameter shows an estimated effect in opposite directions. But in any case, survival time gained beyond “delayed transplant” does not outweigh the disadvantage posed by initial dialysis, where the order of magnitude of time lost under initial dialysis is 5‐fold. In the absence of measured time‐varying confounders, usually required for deriving as‐treated effects, the approach here developed provides empirical evidence for decision making applicable to other settings.

Looking at the applied literature, we found it largely ignores many fundamental statistical lessons learned. This greatly hampers interpretability in our setting and a fortiori transportability to new settings or the relevance of meta‐analyses.^53^ We hope the case study here developed will help support a change in statistical practice and inspire further research on challenges encountered. Below, we respond to some arguments often raised to justify suboptimal analysis and critically reflect on remaining challenges when targeting more explicit causal effect estimation as we did.

Researchers (and editors) see no need to “refine” the approach since the large positive outcome difference for PKT leaves ample room for error before qualitative conclusions change. We found, however, that restriction to the subset of dialysis starters selected upon delayed transplantation, virtually annuls the large survival difference for the full population of dialysis starters (Figure 4). Moreover, with higher risk profiles for kidney transplantation over calendar time, we must anticipate future study populations with smaller magnitudes of effect.

Another argument against careful “causal” analysis is its complexity which might deprive clinicians from a critical understanding of the opportunities and risks involved. We agree that several assumptions play a key role and must be discussed with clinicians. We found the tool of trial emulation, to enhance both insight in the data structure and interpretability of results for a broader scientific audience. Understanding association is clearly more simple but suffers when one jumps all too easily to causal conclusions. As *the critical assumption of NUCB* is fundamentally untestable, derived causal effect estimates should be complemented with an analysis of their sensitivity to various plausible violations. To this end, the helpful concept of an e‐value deserves further development in the survival setting.^54^ We may indeed have missed relevant confounders that account for part of the observed difference in survival between treatment groups as we discuss next.

For our study, comorbidities and kidney function were only available from 1998 and 2008 onward, respectively. Notwithstanding their significant effect on the patient specific hazards (Appendix A), in these cohorts the population average survival remained essentially unchanged with or without adjustment for the newly available variables.

We lacked socio‐economic factors, while patients with higher socio‐economic status could have better access to health care and timely treatment with higher chances of receiving PKT. If so, our estimated PKT advantage remains confounded by socio‐economic factors and may be overestimated. Additional unmeasured confounders may include: unmeasured transient comorbidities such as current infections possibly delaying transplantation, patient preferences or availability of a live donor.^36^, ^55^ The advantage of the Swedish system of registries with a unique patient identifier is that additional variables can be obtained in the future from further linkage.

Focusing on the *ITT effect* of PKT vs dialysis first, we averaged over observed follow‐up strategies as currently implemented. The PKT group then covers the “natural” mix of cadaveric and living donor kidneys. To evaluate the impact on survival of donor type (and other treatment refinements) one could treat “PKT from a living donor” as the specific treatment of interest and study its benefit along the lines established in this article.^8^

Potential survival under dialysis first as estimated from the original models barely changed when using models ignoring covariates needing imputation. Potential survival under PKT however did change, particularly in the observed dialysis first and full RRT group. By including comorbidities, we accounted for worse baseline prognosis in the dialysis first group and reduced the estimated benefit under PKT. Even so, PKT predicted better survival in both the PKT and dialysis first treated. With long term registers that gather periodic information across different centers, there is an unavoidable risk of missing data that may have influenced the estimated effect. It is good practice to enter new patient characteristics in an established registry when their role becomes apparent, or when new diagnostic tools or treatment options are introduced. Considering the importance of incident disease registries as a resource for research providing real world evidence, we feel collaborative efforts should aim to define a minimum set of confounders to be reported. This will enhance transportability of results derived from single registry‐based studies and improve evidence synthesis in meta‐analytic approaches.

For our case study, we included all available confounders considered clinically relevant. Notwithstanding significant contributions to the Cox model, the impact of some covariates on the standardized curves was limited, as shown by the sensitivity analyses. In settings where variable selection is considered, additional steps are needed to derive causal effect estimates. Model building may then entail machine learning and cross‐validation.^56^

In this article, we have dissected opportunities and pitfalls arising when drawing real world evidence on the effect of point exposures on survival from incident disease registers. The nature of these registers enables avoidance of immortal time bias through trial emulation. They can be rich in baseline covariate information, especially when linked with additional registers, but typically lack regular time‐varying covariates. Long term follow‐up of the end point may be needed to inform on relevant patient horizons following treatment decisions. This often comes, however, with a moving target of patient cohorts entering over calendar time. It qualifies attainable estimands which must describe their study population well to allow for transportability with and without extrapolation into the future. It also impacts on assumptions and hence on the choice of causal inference for (asymptotically) unbiased estimates of well‐chosen estimands. We found IP‐weighted KM curves producing biased comparisons due to informative censoring. Standardization through outcome regression avoided this and revealed calendar time trends contributing to the informative nature of administrative censoring. The large differences seen in the case study on PKT vs dialysis first at ESRD, comes with a plea to be specific about study and target population(s) when reporting results and conclusions. This is no less important in subject matter journals which all too often remain fuzzy on such critical points. The relevance is not restricted to long term disease registers, but equally enters when analyzing shorter term survival from cohorts with fast changing populations as in an emerging pandemic.

## AUTHOR CONTRIBUTIONS

Camila Olarte Parra contributed in the conception and design of the study, conducted and interpreted the analysis, and drafted the article. Ingeborg Waernbaum, Staffan Schön, and Els Goetghebeur contributed in the conception and design of the study, interpretation of findings, and revision of the article. All authors approved the final version.

## CONFLICT OF INTEREST

The authors declare no potential conflict of interests.

## FUNDING INFORMATION

This project has received funding from the European Union's Horizon 2020 Research and Innovation Programme under the Marie Sklodowska‐Curie Grant Agreement No. 676207. Ingeborg Waernbaum was funded by the Swedish Research Council, Grant No. 2016‐00703.

## Figures and Tables

**FIGURE A1:**
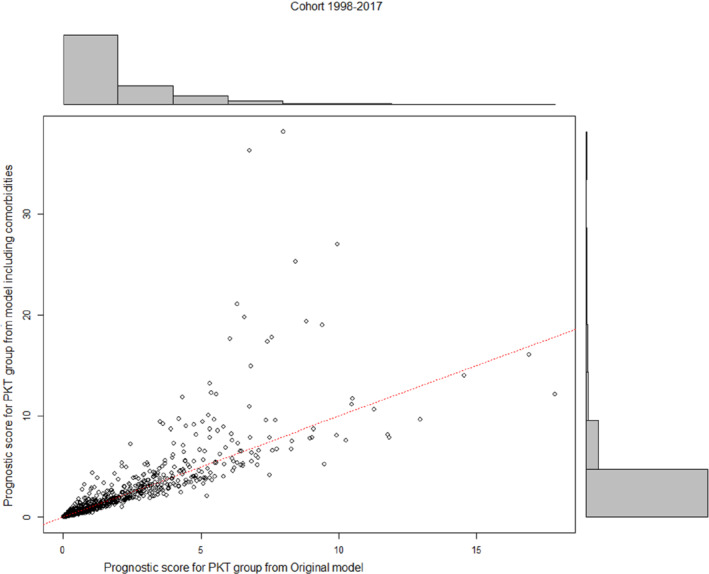
Scatterplot with prognostic scores of the PKT patients as estimated from model B, which includes comorbidities, vs the original model A

**FIGURE A2:**
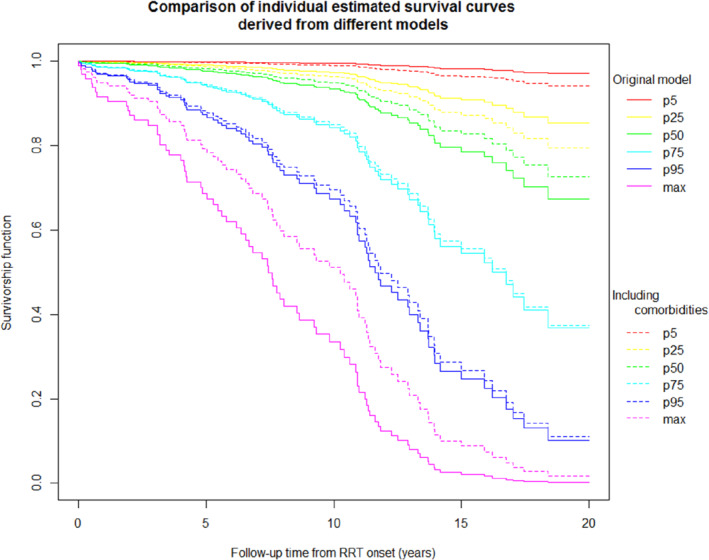
Estimated survival curves derived from the original model A (solid line) compared to model B that adds comorbidities (dashed line) for risk profiles at p5, p25, p50, p75, p95, and the maximum prognostic score for each model for the cohort 1998 to 2017

## Data Availability

The data underlying this article were provided by the Swedish Renal Registry. The use of the data for this study was approved by the Steering Committee of the SRR and by the Ethical Review Board (registration number 2017/305‐31). The code used for all the analyses presented in this article is available at https://github.com/colartep/Causal_PKT.

## APPENDIX A ADDITIONAL FIGURES

### A.1

Below we analyze the subcohort of patients starting RRT from 1998 onwards to illustrate how control for comorbidity indicators affects covariate‐specific survival while it leaves the population average survival virtually unchanged. Figure A1 plots individual prognostic scores in the PKT group based on model B (with indicators for diabetes, hypertension and cardiovascular disease in addition to the original covariates age, sex, kidney disease, and calendar year), vs model A with just the original covariate set. Figure A2 shows (Cox model) derived survival curves at observed percentiles p5, p25, p50, p75, p95, and the maximum. For low risk profiles p5 and p25, reduced model A overestimates survival compared to model B. The opposite happens for higher risk profiles p50 and beyond, where the original model underestimates survival. Ignoring comorbidities thus shows substantial impact on estimated covariate specific survival curves, with negligible effect on the population average survival in the PKT population (Figure A3).
